# Continuous subcutaneous insulin infusion versus multiple daily injection therapy in pregnant women with type 1 diabetes

**DOI:** 10.1111/1753-0407.13558

**Published:** 2024-04-25

**Authors:** Yixin Gong, Tian Wei, Yujie Liu, Jing Wang, Jinhua Yan, Daizhi Yang, Sihui Luo, Jianping Weng, Xueying Zheng

**Affiliations:** ^1^ Department of Endocrinology, The First Affiliated Hospital of USTC, Division of Life Sciences and Medicine University of Science and Technology of China Hefei China; ^2^ School of Medicine Southeast University Nanjing China; ^3^ Department of Obstetrics and Gynecology, The First Affiliated Hospital of USTC University of Science and Technology of China Hefei China; ^4^ Department of Endocrinology and Metabolism The Third Affiliated Hospital of Sun Yat‐sen University Guangzhou China

**Keywords:** continuous subcutaneous insulin infusion, glycemic control, multiple daily injection, pregnancy outcomes, type 1 diabetes

## Abstract

**Introduction:**

The study aimed to compare glycemic control and pregnancy outcomes in women with type 1 diabetes mellitus (T1DM) using multiple daily injection therapy (MDI) and continuous subcutaneous insulin infusion (CSII) and to compare outcomes of women treated with long‐acting insulin or neutral protamine Hagedorn (NPH).

**Methods:**

This multicenter prospective cohort study involved women with pregestational T1DM treated with MDI and CSII. Primary outcome was glycated hemoglobin (HbA1c) before and during pregnancy. Secondary outcomes included maternal and neonatal outcomes and quality of life.

**Results:**

Of the 121 studied women, the average age was 28.48 years, and the average body mass index was 21.29 kg/m^2^ at conception and 26.32 kg/m^2^ at delivery. Of the studied women, 78.51% had planned pregnancy. Women treated with MDI and CSII had comparable HbA1c before pregnancy or in the first and second trimesters. In the third trimester, women on CSII therapy had significantly lower HbA1c (6.07 ± 0.62 vs 6.20 ± 0.88%, *p* = .017), higher HbA1c on‐target rate (71.43% vs 64.62%, *p* = .030), and greater decline of HbA1c from preconception to the third trimester (−0.65 vs −0.30%, *p* = .047). Fewer daily insulin requirements were observed in those used CSII compared with MDI‐treated women (0.60 ± 0.22 vs 0.73 ± 0.25 U/kg/day, *p* = .004). Newborns born of mothers treated with the CSII method were more likely to have neonatal jaundice (adjusted odds ratio [OR] 2.76, 95% confidence interval [CI] 1.16–6.57) and neonatal intensive care unit (adjusted OR 3.73, 95%CI 1.24–11.16), and women on CSII had lower scores in patient‐reported quality of life (*p* = .045). In the MDI group, those receiving long‐acting insulin had nonsignificant lower HbA1c and higher HbA1c on‐target rate in the second and third trimesters, compared with those treated with NPH.

**Conclusions:**

Insulin pump users may achieve better glycemic control than multiple daily insulin injections, which did not substantially improve pregnancy outcome.

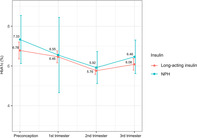

## Graphical abstract

Highlights

- This study is the first to evaluate the effects of multiple daily injection and continuous subcutaneous insulin infusion therapy among Chinese pregnant women with type 1 diabetes.
- We found that pregnant women with type 1 diabetes on continuous subcutaneous insulin infusion therapy had better glycemic control in the third trimester than those on multiple daily injection therapy.
- Continuous subcutaneous insulin infusion users had a significant decline in glycated hemoglobin (HbA1c) level from preconception to the third trimester.
- There was a nonsignificant lower HbA1c level for women treated with long‐acting insulin, compared with neutral protamine Hagedorn.

## 1 INTRODUCTION

Women with pregestational type 1 diabetes mellitus (T1DM) have increased risks of adverse pregnancy outcomes.^1^ Maintaining maternal glucose levels within a target range is a main strategy to reduce the risk of obstetric and neonatal complications.^2^ Multiple daily injection therapy (MDI), known as basal‐bolus regimen (once or twice daily injections of basal insulin combined with rapid‐acting insulin analogs at mealtimes), is recommended by several national guidelines as the therapeutic strategy for T1DM in pregnancy.^3^, ^4^ Continuous subcutaneous insulin infusion (CSII) or pump therapy has shown advantages in glycemic control, reducing episodes of severe hypoglycemia^5^ and leading to a more flexible lifestyle.^6^, ^7^ However, trials in women with pregestational diabetes have shown little agreement on the effect of CSII versus MDI on glycemic control and pregnancy outcomes. Some early studies in the 1980s generally showed CSII not superior to MDI in glycemic control; however, the type of diabetes was uncertain, and technologies on CSII have improved over time.^8^, ^9^, ^10^ More recently, a prespecified secondary analysis of the Continuous Glucose Monitoring in Women With Type 1 Diabetes in Pregnancy Trial (CONCEPTT) has shown that women on MDI therapy had better glycemic control and lower rates of gestational hypertension, neonatal hypoglycemia, and neonatal intensive care unit (NICU) admissions.^11^ In contrast, in a large observational study in Poland involving 209 Caucasian women with T1DM, CSII users achieved a substantially better glycemic control.^12^ Similar findings were also observed in some recent trials showing that CSII or pump therapy enhances metabolic control.^13^, ^14^, ^15^, ^16^ Surprisingly, the expected improvement in pregnancy outcomes in parallel with improved glycemic control is equivocal. These studies showed comparable pregnancy outcomes for pump users despite better^13^, ^14^, ^15^, ^16^ or equivalent glycemic control^17^, ^18^, ^19^ than MDI treatment. Notably, previous studies were generally retrospective studies in which data might be confounded by unknown factors such as education level, preconception diabetic complications, or possibly inadequate metabolic control in pregnancy. In addition, the trials discussed did not specify information on basal insulin regimens such as long‐acting insulin or neutral protamine Hagedorn (NPH).

The aim of the current study was to compare glycemic control and pregnancy outcomes of MDI versus CSII in the pregnant T1DM population. In women treated with MDI, we further explored the effect of insulin type on glycemic control and pregnancy outcomes.

## 2 METHODS

### 2.1 Study design and population

This study was based on the multicenter prospective observational cohort of women with pregestational T1DM from 11 participating centers from 2015 to 2017 in China. A full description of the study cohort has been published previously.^20^ A total of 133 pregnant women aged 20–38 years with T1DM were recruited, and 73.68% (98/133) were planned pregnancy and received preconception care. The selection criteria were those with a single pregnancy, completed follow‐up visits in all trimesters, and recorded obstetric outcomes. The exclusion criteria were individuals unable to be clearly classified as having type 1 or type 2 diabetes, multiple pregnancies and termination of pregnancy due to nonmedical reasons. We further excluded those who take twice daily injections of premixed insulin, take only multiple injections of short or quick‐acting insulin, or take a single dose of basic insulin. Four women who had converted insulin schemes during pregnancy were excluded. A total of 121 women were eligible for final analysis, among those, 65 patients were treated with basal‐bolus MDI and 56 with CSII.

During the whole observational period, all the women received structured care under the framework following the World Health Organization guidance. The trained medical team consisted of an endocrinologist, a diabetes educator, and a dietician providing preconception‐to‐pregnancy management. Every pregnant woman received an individual antenatal care record book at their first antenatal visit. Demographic information was collected including maternal age at delivery, age at T1DM diagnosis, health insurance, the proportion of diabetic costs to household income, education, parity, height, weight, preconception chronic hypertension, preconception diabetic microvascular complications, and episodes of severe hypoglycemia before pregnancy. A checklist covered items on carbohydrate counting, self‐monitoring blood glucose ≥ 7 times per day (recommended glycemic targets during pregnancy were premeal, bedtime, and overnight fingertip glucose 3.3–5.4 mmol/L; peak postprandial fingertip glucose 5.4–7.1 mmol/L), testing glycated hemoglobin (HbA1c) every two months (HbA1c >10% were advised not to get pregnant during prepregnancy care phase; HbA1c targets in the second and third trimester were <6.5%), advice on light and moderate aerobic exercise, the importance of preventing hypoglycemia, and warning signs to go to the clinic visit. The use of continuous glucose monitoring system (CGMS) throughout pregnancy was also recommended on the checklist. The antenatal care record book documented information on maternal anthropometry, laboratory and other test results at an endocrinologist's or an obstetrician's discretion. We also used the electronic medical record system at the participating center as a source of information on any hospitalization or episodes of diabetic ketoacidosis (DKA) during pregnancy.

The decision on insulin mode was made by an endocrinologist after a detailed discussion with the patient with regard to their preference and evaluation of the patient's ability to properly use the CSII. Insulin dose adjustments according to blood glucose were made at each prenatal visit by an endocrinologist. Insulin aspart was used for those receiving CSII, whereas for MDI, basal‐bolus schemes of detemir, glargine, or NPH were used as basal insulin and insulin aspart as the short‐acting insulin before meals. Both MDI and CSII users received the same diabetes self‐management education in each participating center. Daily dose of insulin (DDI) was calculated with daily total insulin (U) divided by weight (kg). All women signed an informed consent form. The study was approved by the Ethics Committee of the Third Affiliated Hospital of Sun Yat‐sen University.

### 2.2 Maternal and neonatal outcomes

Information on pregnancy outcomes was collected including (a) maternal outcomes–preeclampsia, gestational hypertension, cesarean section, progression of diabetic complications (diabetes nephropathy, diabetes retinopathy); excessive gestational weight gain; DKA; and feeding mode. Preeclampsia is defined as systolic blood pressure of ≥140 mm Hg and/or diastolic blood pressure of ≥90 mm Hg accompanied by urinary proteinuria of ≥ + or without proteinuria but accompanied by terminal organ disease. The progression of diabetic nephropathy in pregnancy is defined as an increase of 26 umol/L or 0.3 mg/dL in serum creatinine from the first trimester to the third trimester, or an increase of 50%–99% in serum creatinine compared with the first trimester. The progress of diabetic retinopathy in pregnancy was based on White's classification of complications of diabetes in pregnancy,^21^ and (b) neonatal outcomes–congenital malformations, preterm birth (gestational age <37 weeks at delivery), macrosomia (birth weight ≥4000 g), large for gestational age (LGA, birth weight higher than the 90th percentile of gestational age), small for gestational age (SGA, birth weight lower than the 10th percentile of gestational age), neonatal hypoglycemia (postnatal capillary blood glucose<2.2 mmol/L), neonatal jaundice (a need for phototherapy or exchange transfusion), Apgar score <8, NICU (admission to a higher‐level neonatal care nursery >24 hours during the initial hospitalization after birth), and neonatal respiratory distress syndrome (RDS).

Patient‐reported outcomes were measured with EuroQoL‐5D (EQ‐5D) questionnaire comprising possible health status on mobility, self‐care, usual activities, pain/discomfort, and anxiety/depression in the first trimester (<12 week), the second trimester (16–24 week), and the third trimester (28–41 week). Those who reported any problem in any dimension of EQ‐5D would be regarded as having impaired quality of life. We also used the Chinese time trade‐off value method to calculate EQ‐5D score.^22^

For further comparison of outcomes regarding the program of insulin treatment, patients were stratified into subgroups of glargine (*n* = 16), detemir (*n* = 35) and NPH (*n* = 10).

### 2.3 Statistical analysis

All statistical analyses were performed using SPSS (version 23.0) and R software (version 4.1.1). Data on maternal characteristics and clinical parameters are presented as means ± SDs or median (interquartile range) for continuous variables and percentages for quantitative variables. Student *t* test, analysis of covariance, Pearson chi‐square test, or Fisher's exact method were used where appropriate. Logistic regression models adjusted for potential covariates were used for qualitative variables, and linear regression models were used for continuous outcomes. Significance was set at a two‐sided *p* value <.05.

## 3 RESULTS

### 3.1 Characteristics of the studied women

Table 1 shows the baseline characteristics of the women with pregestational T1DM treated with CSII or MDI. Of the 121 studied women (65 used MDI and 56 used CSII during pregnancy), 95 (78.51%) were planned pregnancies and received prepregnancy care. The mean maternal age was 28.48 ± 3.72 years, with the median duration of diabetes of 7.0 (2.0,11.5) years. The mean body mass index (BMI) was 21.29 ± 2.71 kg/m^2^ at conception and 26.32 ± 2.84 kg/m^2^ at delivery. The proportion of women using CGMS was comparable between the two groups (76.92% and 80.36%, *p* = .753). We did not observe significant differences in maternal age, health insurance, proportion of diabetic costs to household income, maternal and husband's education level, parity, maternal BMI, preconception diabetic microvascular complications, and episodes of preconception severe hypoglycemia between the two groups. Women using CSII had a longer duration of diabetes (*p* = .012) and were more likely to represent class C and class D of the White classification (*p* = .035) than the MDI users. More women treated with CSII received prepregnancy care (*p* = 0.035). Women on CSII therapy had a significantly reduced daily insulin requirement (0.60 ± 0.22 U/kg/day) compared with MDI users (0.73 ± 0.25 U/kg/day, *p* = .004) at 37 gestation weeks. Besides, the basal to total insulin was elevated in patients treated with CSII (p < .001).

**TABLE 1:** Baseline characteristics of pregnant women with T1DM using MDI and CSII therapy.

Variable	MDI (*n* = 65)	CSII (*n* = 56)	*p*
Age (years)	28.09 ± 3.77	28.95 ± 3.67	.201
Duration of diabetes, years	5 (2.9)	9.5 (3.15)	.012
Health insurance, *n* (%)	57 (87.69)	54 (96.43)	.072
Proportion of diabetes cost to household income, %	10 (5.20)	10 (7.75.23.75)	.367
Education level, *n* (%)			.126
High school or lower	18 (27.69)	9 (16.07)	
College or higher	47 (72.31)	47 (83.93)	
Husband's education, *n* (%)			.141
High school or lower	16 (24.62)	5 (8.93)	
College or higher	49 (75.38)	51 (91.07)	
Nulliparous, *n* (%)	53 (81.54)	45 (80.36)	.869
Prepregnancy care, *n* (%)			
Yes	46 (70.77)	49 (87.50)	.035
No	18 (27.69)	7 (12.5)	
Maternal body weight			
Prepregnancy BMI, kg/m^2^	21.23 ± 2.38	21.35 ± 2.99	.844
BMI at 37 weeks, kg/m^2^	26.05 ± 2.66	26.60 ± 3.02	.321
Chronic hypertension, *n* (%)	0	3 (5.36)	.104
Hyperlipidemia, *n* (%)	3 (4.62)	4 (7.14)	.584
Thyroid disease, *n* (%)	15 (23.08)	16 (28.57)	.560
Preconception diabetic microvascular complications, *n* (%)			
Diabetes nephropathy	2 (3.08)	3 (5.36)	.530
Diabetes retinopathy	2 (3.08)	4 (7.14)	.302
Preconception severe hypoglycemia, *n* (%)	22 (33.85)	21 (37.50)	.704
White classification			.035
A Sufficient glycemic control with diet alone	0	0	
B Age of onset >20 years old, and duration <10 years	43 (66.15)	27 (48.21)	
C Age of onset 10–19 years old, and duration 10–19 years	21 (32.31)	23 (41.07)	
D Age of onset <10 years old or duration ≥20 years or retinopathy	1 (1.54)	6 (10.71)	
Used CGMS, *n* (%)	50 (76.92)	45 (80.36)	.753
DDI at 37 weeks, U/kg/day	0.73 ± 0.25	0.60 ± 0.22	.004
Basal rate at 37 weeks, *n* (%)	0.31 ± 0.11	0.42 ± 0.14	<.001

*Note*: Data are presented as mean ± SD, median (interquartile range) or N (percentage).

Abbreviations: BMI, body mass index; CGMS, continuous glucose monitoring system; CSII, continuous subcutaneous insulin infusion; DDI, daily dose of insulin; MDI, multiple daily injection; T1DM, type 1 diabetes mellitus.

### 3.2 Comparison of diabetes parameters

Comparisons on glycemic control were made with HbA1c‐related parameters rather than CGM‐derived parameters in the current study due to the limited period of CGM use. HbA1c from preconception to the third trimester are presented in Table 2. Preconception HbA1c level and proportion of preconception HbA1c on‐target rate were similar between women on MDI and CSII therapy (*p* = 0.173). In the first and second trimesters, women using CSII had nonsignificant lower HbA1c levels than those using MDI (*p* > .05). Glycemic control in both groups improved over time. HbA1c was significantly lower for CSII users in the third trimester (*p* = .017) and more CSII users achieved the targeted HbA1c level of <6.5% in the third trimester (71.43% vs 64.62%, *p* = .03). The decrease in HbA1c levels between the two groups from preconception to the third trimester differs between the MDI and CSII users (−0.30 [−1.00, 0.25] vs −0.65 [−1.19, −0.38], *p* = .047).

**TABLE 2:** Glycemic outcomes of women using MDI and CSII during pregnancy.

Glycemic outcomes	MDI (*n* = 65)	CSII (*n* = 56)	Unadjusted *p*	Adjusted *p*
Preconception HbA1c				
Mean ± SD, %	6.90 ± 1.31	7.10 ± 1.27	.399	.173
Mean ± SD, mmol/mol	51.92 ± 14.28	54.09 ± 13.76		
HbA1c < 6.5% or 48 mmol/mol, *n* (%)	32 (49.23)	19 (33.93)	.084	.052
HbA1c in the first trimester				
Mean ± SD, %	6.59 ± 1.09	6.56 ± 0.87	.868	.807
Mean ± SD, mmol/mol	48.40 ± 11.94	48.20 ± 9.58		
HbA1c < 6.5% or 48 mmol/mol, *n* (%)	30 (46.15)	31 (55.36)	.408	.403
HbA1c in the second trimester				
Mean ± SD, %	5.85 ± 0.66	5.75 ± 0.49	.260	.281
Mean ± SD, mmol/mol	40.43 ± 7.22	39.30 ± 5.28		
HbA1c < 6.5% or 48 mmol/mol, *n* (%)	35 (53.85)	39 (69.64)	.208	.281
HbA1c in the third trimester				
Mean ± SD, %	6.20 ± 0.88	6.07 ± 0.62	.012	.017
Mean ± SD, mmol/mol	44.20 ± 9.51	42.89 ± 6.80		
HbA1c < 6.5% or 48 mmol/mol, *n* (%)	42 (64.62)	40 (71.43)	.023	.030
Change from preconception to the third trimester				
Median (IQR), %	−0.30 (−1.00, 0.25)	−0.65 (−1.19, −0.38)	.039	.047
Median (IQR), mmol/mol	−4.64 (−17.35, 0.96)	−8.20 (−14.75, −4.37)		

*Note*: Model adjusted for maternal age, duration of diabetes, BMI, and daily dose of insulin.

Abbreviations: BMI, body mass index; CSII, continuous subcutaneous insulin infusion; HbA1c, glycated hemoglobin; IQR, interquartile range; MDI, multiple daily injection.

### 3.3 Pregnancy outcomes

Pregnancy outcomes in women on MDI and CSII therapy are summarized in Table 3. After adjusting for maternal age, duration of diabetes, BMI, and daily dose of insulin, CSII therapy was associated with elevated risks of neonatal jaundice (adjusted odds ratio [aOR] 2.76, 95% confidence interval [CI] 1.16–6.57), and NICU (aOR 3.73, 95% CI 1.24–11.16), compared with the MDI group. There was no significant difference between the two groups on the incidence of preeclampsia, gestational hypertension, cesarean section, progression in diabetic complications, excessive gestational weight gain, DKA, breastfeeding, congenital malformation, preterm birth, early preterm birth, macrosomia, LGA, SGA, neonatal hypoglycemia, Apgar score <8, and RDS (all *p* > .05).

**TABLE 3:** Comparison of pregnancy outcomes between MDI and CSII groups.

Pregnancy outcomes	MDI (*n*= 65)	CSII (*n* = 56)	*p*	aOR 95%CI
Maternal outcomes, *n* (%)				
Preeclampsia	4 (6.15)	3 (5.36)	0.852	0.86 (0.19–4.03)
Gestational hypertension	4 (6.15)	7 (12.50)	0.235	2.18 (0.60–7.87)
Cesarean section	38 (58.46)	39 (69.64)	0.188	1.78 (0.83–3.82)
Progression in diabetic complications	2 (3.08)	7 (12.50)	0.058	4.88 (0.95–25.15)
Diabetic nephropathy	1 (1.54)	4 (7.14)	0.177	4.62 (0.50–42.60)
Diabetic retinopathy	1 (1.54)	4 (7.14)	0.183	4.53 (0.49–41.79)
Gestational weight gain, kg	12.50 ± 5.44	12.96 ± 4.62	0.676	0.51 (−1.67–2.68)
Excessive gestational weight gain	17 (26.15)	14 (25.00)	0.971	0.98 (0.37–2.62)
DKA, *n* (%)	0	1 (1.79)	0.467	NA
Breastfeeding, *n* (%)	35 (53.84)	40 (71.43)	0.050	2.18 (0.99–4.77)
Neonatal outcomes, *n* (%)				
Congenital malformation	4 (6.15)	3 (5.36)	0.854	0.87 (0.18–4.05)
Preterm birth	10 (15.38)	8 (14.29)	0.800	0.88 (0.32–2.43)
Early preterm birth (24–30 weeks)	2 (3.08)	1 (1.79)	0.770	0.69 (0.06–8.13)
Macrosomia	6 (9.23)	2 (3.57)	0.214	0.35 (0.67–1.83)
LGA	9 (13.85)	7 (12.50)	0.761	0.80 (0.19–3.36)
SGA	7 (10.77)	1 (1.79)	0.065	0.15 (0.02–1.24)
Neonatal hypoglycemia	7 (10.77)	8 (14.29)	0.249	1.42 (0.48–4.20)
Neonatal jaundice	11 (16.92)	21 (37.50)	**0.022**	**2.76 (1.16**–**6.57)**
Apgar score <8	4 (6.15)	5 (8.93)	0.485	1.74 (0.37–8.26)
NICU	13 (20)	21 (37.50)	**0.019**	**3.73 (1.24**–**11.16)**
RDS	3 (4.62)	3 (5.36)	0.867	1.15 (0.22–5.95)

*Note*: Analysis were adjusted for maternal age, duration of diabetes, BMI, and daily dose of insulin. Variables with statistical significance were shown in boldface.

Abbreviations: BMI, body mass index; CI, confidence interval; CSII, continuous subcutaneous insulin infusion; DKA, diabetic ketoacidosis; LGA, large for gestational age; aOR, adjusted odds ratio; MDI, multiple daily injection; NICU, neonatal intensive care unit; RDS, respiratory distress syndrome; SGA, small for gestational age.

### 3.4 Secondary outcomes

**FIGURE 1:**
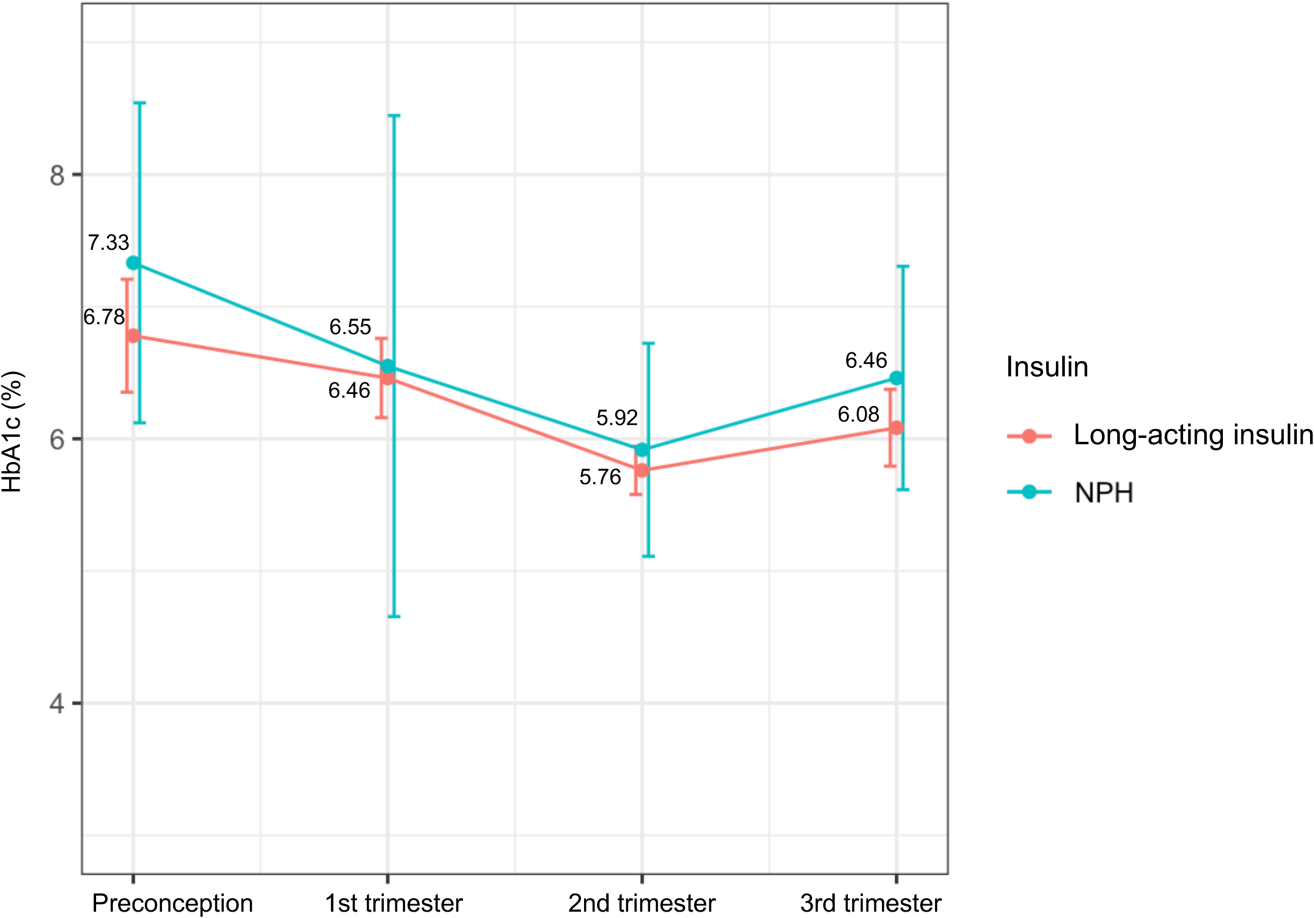
HbA1c change throughout gestation period among MDI users treated with long‐acting insulin or NPH. HbA1c, glycated hemoglobin; MDI, multiple daily injection therapy; NPH, neutral protamine Hagedorn.

In the subgroup analysis, 61 eligible data for MDI users were stratified by basal insulin of glargine (*n* = 16), detemir (*n*= 35), and NPH (*n* = 10). The three groups had no significant difference in glycemic control throughout gestation. NPH users tend to have higher HbA1c than long‐acting insulin (glargine/detemir) users in the second and third trimesters, the differences were not significant (Table 4, Figure 1). Also, maternal and fetal outcomes were similar among groups of different basal insulin types (Table S1).

**TABLE 4:** Comparison of glycemic outcomes between MDI users treated with insulin glargine, detemir, and NPH.

Glycemic outcomes	Glargine (*n* = 16)	Detemir (*n* = 35)	NPH (*n* = 10)	NPH compared–long‐acting insulins (detemir /glargine)		Detemir compared–glargine		Compared–NPH			
								Detemir		Glargine	
				aOR (95% CI)	*p*	aOR (95% CI)	*p*	aOR (95% CI)	*p*	aOR (95% CI)	*P*
Preconception HbA1c (%)	6.66 ± 1.59	6.82 ± 1.21	7.23 ± 1.39	0.22 (−0.27–0.72)	0.363	0.17 (−0.82–1.15)	0.735	0.41 (−0.57–1.38)	0.406	0.29 (−0.44–1.01)	0.418
Preconception HbA1c < 6.5% or 48 mmol/mol (n (%))	6 (37.50)	20 (57.14)	4 (40.00)	0.85 (0.41–1.76)	0.659	2.00 (0.46–8.70)	0.355	0.60 (1.32–2.72)	0.508	1.10 (0.44–2.73)	0.845
HbA1c in the first trimester (%)	6.56 ± 1.21	6.41 ± 0.86	6.57 ± 1.81	0.14 (−0.31–0.59)	0.530	−0.15 (−0.79–0.50)	0.650	0.33 (−0.58–1.24)	0.465	0.33 (−0.58–1.24)	0.465
First trimester HbA1c < 6.5% or 48 mmol/mol (*n* (%))	6 (37.50)	20 (57.14)	6 (60.00)	1.13 (0.50–2.53)	0.769	2.55 (0.68–9.54)	0.164	0.94 (1.78–5.00)	0.943	1.55 (0.61–3.91)	0.355
HbA1c in the second trimester (%)	5.66 ± 0.58	5.80 ± 0.53	6.21 ± 1.05	0.23 (−0.04–0.49)	0.094	0.14 (−0.27–0.55)	0.493	0.41 (−0.16–0.99)	0.153	0.41 (−0.16–0.99)	0.153
Second trimesterHbA1c < 6.5% or 48 mmol/mol (*n* (%))	10 (62.50)	21 (60)	4 (40.00)	0.69 (0.30–1.57)	0.377	1.07 (0.24–4.74)	0.932	0.47 (0.09–2.55)	0.380	0.71 (0.27–1.88)	0.488
HbA1c in the third trimester (%)	6.22 ± 1.08	6.03 ± 0.79	6.46 ± 0.68	0.19 (−0.22–0.60)	0.355	−0.19 (−0.85–0.47)	0.560	0.43 (−0.33–1.19)	0.259	0.12 (−0.46–0.70)	0.662
Third trimester HbA1c < 6.5% or 48 mmol/mol (*n* (%))	8 (50.00)	13 (37.14)	2 (20.00)	0.59 (0.18–1.86)	0.369	0.63 (0.14–2.72)	0.531	0.40 (0.04–4.11)	0.441	0.50 (0.14–1.76)	0.280
Change from preconception to the third trimester (%)	−0.37 (−1.43, 0.09)	−0.70 (−1.38, −0.15)	−1.28 (−2.33, −0.33)	0.08 (−0.48–0.65)	0.771	0.35 (−0.58–1.28)	0.448	0.06 (−1.08–1.20)	0.914	0.21 (−0.60–1.01)	0.594

*Note*: Data are presented as mean ± SD, median (interquartile range) or N (percentage).Analysis were adjusted for maternal age, duration of diabetes, BMI, and daily dose of insulin.

Abbreviations: aOR, adjusted odds ratio; BMI, body mass index; CI, confidence interval; HbA1c, glycated hemoglobin; MDI, multiple daily injection; NPH, neutral protamine Hagedorn.

Women in the CSII group had decreased quality of life with a significantly lower mean EQ‐5L score than MDI users (1[0.875, 1] vs 0.875[0.869, 1], *p* = .045). More CSII users reported problems in EQ‐5D estimated quality of life during pregnancy than the MDI‐treated group (46.94% vs 28.33%, *p* = .045, Table 5).

**TABLE 5:** EQ‐5D estimated quality of life of women using CSII and MDI.

Dimension	First trimester		Second trimester		Third trimester		Throughout pregnancy		*F/χ* ^2^	*p*
	MDI	CSII	MDI	CSII	MDI	CSII	MDI	CSII		
*N* (%)	49	41	42	37	35	31	60	49		
1 Mobility	0	1 (2.44)	6 (14.29)	4 (10.81)	0	1 (3.23)	6 (10)	5 (10.20)	0.008	.930
2 Self‐care	0	0	3 (7.14)	2 (5.41)	0	0	3 (5)	2 (4.08)	0.095	.758
3 Daily activities	0	2 (4.88)	2 (4.76)	2 (5.41)	0	1 (3.23)	2 (3.33)	3 (6.12)	0.369	.664
4 Pain/discomfort	3 (6.12)	2 (4.88)	10 (23.81)	9 (24.32)	3 (8.57)	6 (19.35)	11 (18.33)	14 (28.57)	1.088	.297
5 Anxiety/depression	8 (16.33)	6 (14.63)	11 (26.19)	9 (24.32)	3 (8.57)	7 (22.58)	16 (26.67)	16 (32.65)	0.187	.665
Quality of life										
No problem	39 (79.59)	33 (80.49)	29 (69.05)	24 (64.86)	29 (82.86)	19 (61.29)	43 (71.67)	26 (53.06)	**4.019**	**.045**
Reported problem	10 (20.41)	8 (19.51)	13 (30.95)	13 (35.14)	6 (17.14)	12 (38.71)	17 (28.33)	23 (46.94)		
EQ‐5D score	N/A	N/A	N/A	N/A	N/A	N/A	1 (0.875,1)	0.875 (0.869,1)	**−2.008**	**.045**

*Note*: Data are presented as N (percentage) or median (interquartile range). Variables with statistical significance were shown in boldface.

Abbreviations: CSII, continuous subcutaneous insulin infusion; EQ‐5D, EuroQoL‐5D questionnaire; MDI, multiple daily injection.

## 4 DISCUSSION

In this observational study of pregnant women with T1DM, we did not find significant differences in HbA1c levels between the CSII and MDI groups before pregnancy or in the first and second trimesters. CSII users had improved glycemic control in the third trimester with a significantly lower HbA1c than MDI‐treated women. The proportion of women using CGMS was comparable between both groups. Previously published studies on the effect of MDI and CSII on glycemic control are not consistent. In the Relative Effectiveness of Pumps Over MDI and Structured Education (REPOSE) trial of the nonpregnant T1DM population, participants randomized to pumps and MDI at baseline had a nonsignificant decline in HbA1c over 24 months with −0.85% for pump users and −0.42 for MDI users.^23^ As for pregnancies, a Swedish cohort of women complicated with T1DM reported similar glucose levels in each trimester for MDI and pump users.^24^ In the CONCEPTT study, in which CGMS were randomized to women with T1DM, MDI users had lower HbA1c level in the second trimester and a greater decline in HbA1c level from randomization to 34 gestation weeks of −0.55%.^11^ In contrast, a Poland retrospective study of 209 Caucasian women showed CSII users achieved a substantially better HbA1c level in the first and second trimesters.^12^ Likewise, the Italian Group for Continuous Subcutaneous Insulin Infusion in Pregnancy (IGCSIIP) study reported that CSII users achieved therapeutic HbA1c targets earlier during pregnancy and had better metabolic control at parturition than MDI.^14^ Notably, pump users had a longer duration of diabetes and were affected more frequently by chronic complications, which has also been observed in our study that CSII users were in a higher White's class at conception.^14^ It can be speculated that patients were likely to choose a more advanced insulin therapy mode if they were classified as in a more challenging condition with higher difficulties in achieving therapeutic targets (eg, longer duration of diabetes, more diabetic complications). We collected information on demographic characteristics and adjusted for potential confounders in the final analysis. Our results showed the effectiveness of CSII in glycemic control regardless of longer duration of diabetes at conception.

In accordance with previous studies involving pregnant T1DM populations showing pump users had equivalent glycemic control but less daily insulin dose/kg,^18^, ^25^, ^26^ we also observed that CSII required a significantly less insulin dosage per kg with lower HbA1c in late pregnancy than MDI. In the IGCSIIP study,^14^ a nonsignificant lower daily insulin dose/kg in CSII users was observed. However, an Italy retrospective study of women complicated by T1DM reported a higher DDI for CSII users to achieve comparable HbA1c levels to MDI users.^17^ The author did not note the possible explanation but they did not adjust for maternal weight in the analysis.

CSII might be helpful in reducing the incidence of hypoglycemia with regard to the advantage of reducing insulin dosage. Indeed, decreased episodes of severe hypoglycemia in CSII users among nonpregnant patients with T1DM were previously reported.^5^, ^27^ As for the pregnant population, a multicenter study in Canada showed that women who used insulin pumps achieved lower HbA1c from the first to the third trimester without increased episodes of severe hypoglycemia during pregnancy.^13^ Along this line, data from CONCEPTT suggested that pump users spend less time in the hypoglycemia range throughout gestation, estimated by CGMS, compared with MDI users.^11^ Although severe hypoglycemia during pregnancy was not counted in our study, the indirect evidence suggested that women who used CSII could have achieved near normal blood glucose without increasing severe hypoglycemia episodes, based on the evidence that severe hypoglycemia before pregnancy was a predisposing factor for severe hypoglycemia during pregnancy and similar episodes of preconception severe hypoglycemia was recorded between groups in the current study.^28^

In addition to severe hypoglycemia, DKA is a potential complication of insulin failure during pregnancy. It is well known that pregnancy, particularly in the last half of pregnancy, due to accelerated catabolism, increases insulin needs and that the greater production of hormones (eg, estrogen, cortisol, and human placental lactogen) contributes to insulin resistance, a condition of increased risk of DKA.^29^ In our study, DKA occurred in one patient in the CSII group, which failed to show statistical significance. Previous studies suggested CSII users may achieve better glycemic control without increasing the risks of DKA.^13^, ^25^ In CONCEPTT, the reported DKA rate was 1.7% in pump users and 2.48% in MDI users.^11^ Only one study from Israel reported an increased incidence of DKA among CSII users,^19^ in which the CSII group was small (30 women) and allocation for insulin mode was based on patient preference, which constitutes a potential confounder regarding lifestyle issues for CSII users. Collectively, it is worth noting the beneficial effect of CSII over MDI in terms of lowering the risk of DKA considering the feasibility of small, precise dose increments and the proven lower dosage of insulin for CSII treatment.

Our findings are aligned with previously reported data that those who received prepregnancy care were more likely to receive CSII treatment.^12^, ^13^, ^26^ A majority of women (95, 78.51%) received prepregnancy care. Factors such as longer duration of diabetes and higher rates of prepregnancy diabetic complications, which might be recognized as high‐risk pregnancies and lead to the initiation of CSII, are by all means clinically relevant issues in the evaluation and decision‐making process in prepregnancy care. Nevertheless, prepregnancy planning has been proven to be an effective strategy for improving glycemic control and pregnancy outcomes in diabetic pregnancies regardless of the mode of insulin delivery.^12^

In our study, CSII‐treated women had higher rates of neonatal jaundice and NICU admission despite the improved glycemic control in the subsets. The results are in agreement with recently published literature suggesting a higher trend toward neonatal complications for pump users.^11^, ^24^ This is unexpected and hard to explain. Although we have done our best to adjust for all available potential confounders, we cannot guarantee that we have eliminated all biases. It is plausible that a more challenging baseline situation can influence both glycemic control and pregnancy outcome. In addition, studies showed that interventions during pregnancy had little impact on neonatal jaundice in diabetic pregnancies, indicating such neonatal complications might emerge in the early stage of embryo development related to uteroplacental underperfusion.^30^ Data concerning birth weight showed no difference in the current study with similar rates of macrosomia, LGA, and SGA. Similar findings have also been reported by the CONCEPTT study.^11^ In addition, we observed similar rates of congenital malformation between two groups. Congenital malformations mostly occur in early pregnancy during organogenesis and are not significantly influenced by factors during pregnancy. Therefore, strategies aiming to reduce neonatal complications should ideally be initiated in early pregnancy. The conflicting results of improved glycemic control and nonsuperior obstetric outcomes underscore the ongoing need for additional approaches to improve maternal and fetal health. Preliminary data suggest that the use of modern technologies such as overnight closed‐loop therapy^31^ and CGMS^32^ was potentially beneficial in pregnant women with T1DM.

It is also worth noting that women on MDI therapy reported a more ideal quality of life during pregnancy. This is consistent with the study by Feig et al,^11^ who also found that MDI users had less decline in self‐rated health and well‐being during pregnancy. In contrast, it has previously been established that pump users tend to have less worry about hypoglycemia and a more flexible lifestyle.^7^, ^11^ Despite the possible confounders on background factors, it is noteworthy that CSII is still a complicated tool in light of so many advantages which requires constant insulin dose modification; thus, resources directed to those CSII users may be beneficial.

The available data concerning the effect of insulin delivery mode on maternal gestational weight gain vary across studies. In the extant literature, higher^25^ and lower^15^ gestational weight gain among pump users in pregnancy was reported. Gestational weight gain indirectly indicates the metabolism‐related risk of maternal and newborn babies. Excessive gestational weight gain may be accompanied by elevated levels of circulating free fatty acids and triglycerides, leading to fetus hyperinsulinemia and, subsequently, excessive growth. We found no differences between the two groups in maternal gestational weight gain, rate of excessive gestational weight gain, or neonatal birth weight. The maternal gestational weight gain of 12.77 kg in our study was comparatively lower than the 13.5 kg in the CONCEPTT trial and 16.3 kg in the Canadian pump study, but higher than 11.55 kg in the IGCSIIP study. Generally, the aforementioned studies were generally cohort studies under strict study design and management, indicating that adequate education, dietary counseling, and weight management during pregnancy are required for women with T1DM.

The results were similar for insulin glargine, insulin detemir, and NPH insulin treatment with respect to all secondary variables evaluated. There was a trend toward lower HbA1c levels in women treated with long‐acting insulin (glargine and detemir) compared with NPH insulin; also, a smaller percentage of women achieved target HbA1c (<6.5%) with NPH in the second and the third trimester, but these differences were not statistically significant. A randomized, controlled trial with pregnant women with T1DM declared detemir (HbA1c 6.27% at 36 gestational weeks) noninferior to NPH (HbA1c 6.33% at 36 gestational weeks).^33^ A study of women with gestational diabetes and type 2 diabetes showed similar glycemic control and obstetric outcomes between detemir and NPH.^34^ The issue of hypoglycemia has been controversial, with some studies showing the advantage of long‐acting insulin in reducing hypoglycemia episodes compared with NPH,^34^, ^35^ whereas in others, the rates of hypoglycemia were comparable.^33^, ^36^, ^37^ More studies are required to better understand the detailed association between insulin type and pregnancy outcomes in women with T1DM.

Our study has several strengths. First, it was a large cohort of pregnant women with T1DM across 11 centers in China with the implementation of a standard comprehensive management plan. Patients in each participating center got standardized treatment and potential confounders were therefore mitigated to the largest extent. Second, a majority of the studied women were planned pregnancies and received prepregnancy care. Third, detailed information regarding preconception chronic diseases, weight gain during pregnancy, and other important confounding variables, such as daily insulin dosage and economic factors (health insurance, proportion of diabetes cost to total income) were all controlled in the current study. The relationship between insulin mode and pregnancy outcomes can therefore be well evaluated. Fourth, the studied women represent a population different from previously reported studies whose participants were younger at conception, had a shorter duration of diabetes, and had lower body weight. There are a few shortcomings in our study. First, women were not randomized to CSII or MDI. Although we made every effort to adjust for potential confounders, it cannot be excluded that both the women's and physicians' preferences may influence their decision and lead to data bias. Second, we do not have data on episodes of hypoglycemia during pregnancy. Noteworthy, the prevention and treatment for hypoglycemia were emphasized in the checklist. Third, glycemic control was assessed only based on HbA1c levels. Although we have recommended using CGM according to guidelines for pregestational diabetes, the number of women adopting CGM was limited due to economic constraints. Fourth, our study is based on a cohort under careful management, which may be less generalizable to all areas, particularly where unmet needs for multidisciplinary care for the pregnant T1DM population remain.

## 5 CONCLUSION

Pregnant women treated with insulin pump had a larger decrease in HbA1c across gestation than multiple daily injections of insulin and required less insulin dosage. Insulin pump is one tool that may assist in achieving the near‐normal glycemic targets in pregnancy; further studies are needed to find strategies to improve pregnancy outcomes for the pregnant T1DM population.

## AUTHOR CONTRIBUTIONS

Yixin Gong and Tian Wei conceived the idea and contributed to data analysis. Yixin Gong wrote the original draft of the manuscript. Sihui Luo and Yujie Liu verified the underlying data reported in the manuscript. Jing Wang contributed to the quality control of the database. Jinhua Yan and Daizhi Yang assisted in the supervision of the project. Jianping Weng is the principal investigator of this study and takes responsibility for the integrity of the data. Xueying Zheng contributed to project management, data interpretation, and critical revision of the manuscript.

## FUNDING INFORMATION

The study was funded by the Strategic Priority Research Program of Chinese Academy of Sciences (grant number XDB38010100), the National Natural Science Foundation of China (grant number 82100822) and the Hefei Comprehensive National Science Center (grant number BJ9100000005).

## DISCLOSURE

The authors declare that they have no known competing financial interests or personal relationships that could have appeared to influence the work reported in this paper.

## Supporting information


**Table S1.** Comparison of pregnancy outcomes between women treated with insulin glargine, detemir, and NPH. NPH, neutral protamine Hagedorn.
