# Supplementary material for: Continuous subcutaneous insulin infusion versus multiple daily injection therapy in pregnant women with type 1 diabetes
Source: J Diabetes. 2024 Apr 25;16(5):e13558. doi: 10.1111/1753-0407.13558 (PMC11045913; doi:10.1111/1753-0407.13558)
Supplement: Supplementary file 1 — Table S1. Comparison of pregnancy outcomes between women treated with insulin glargine, detemir, and NPH. NPH, neutral protamine Hagedorn. [file JDB-16-e13558-s001.docx]

Table S1 Comparison of pregnancy outcomes between women treated with insulin glargine, detemir, and NPH

|  | Analogs-glargine (n=16) | Analogs-detemir (n=35) | Analogs-NPH  (n=10) | Analogs- NPH compared to long-acting insulins (detemir /glargine) | | Analogs- detemir compared to Analogs- glargine | | Compared to Analogs- NPH | | | |
| --- | --- | --- | --- | --- | --- | --- | --- | --- | --- | --- | --- |
|  |  |  |  |  |  |  |  | Analogs- detemir | | Analogs- glargine | |
|  |  |  |  | aOR (95% CI) | *P* | aOR (95% CI) | *P* | aOR (95% CI) | *P* | aOR (95% CI) | *P* |
| **Maternal outcomes** |  |  |  |  |  |  |  |  |  |  |  |
| Gestational weigh gain | 11.71±3.19 | 12.63±6.26 | 12.80±4.92 | 0.22(-2.43 to 2.86) | 0.870 | 0.92(-3.33 to 5.18) | 0.662 | 0.17(-5.94 to 6.27) | 0.955 | 0.54(-1.71 to 2.80) | 0.610 |
| Excessive gestational weight gain | 0 | 9(25.71) | 2(20.00) | 0.82(0.26 to 2.61) | 0.732 | N/A |  | 0.39(0.04 to 4.06) | 0.430 | N/A | N/A |
| Preeclampsia | 1(6.25) | 2(5.71) | 1(10.00) | 1.33(0.41 to 4.37) | 0.635 | 0.91(0.08 to 10.82) | 0.940 | 1.83(0.15 to 22.58) | 0.636 | 1.29(0.30 to 5.48) | 0.729 |
| Hypertension in pregnancy | 1(6.25) | 2(5.71) | 1(10.00) | 1.33(0.41 to 4.37) | 0.635 | 0.91(0.08 to 10.82) | 0.940 | 1.83(0.15 to 22.58) | 0.636 | 1.29(0.30 to 5.48) | 0.729 |
| Cesarean section | 8(50.00) | 20(57.14) | 7(70.00) | 1.35(0.65 to 2.81) | 0.417 | 1.43(0.43 to 4.72) | 0.558 | 1.63(0.36 to 7.43) | 0.526 | 1.53(0.66 to 3.52) | 0.320 |
| Progression in diabetic microvascular disease | 1(6.25) | 1(2.86) | 0 | N/A | N/A | 0.44(0.03 to 7.53) | 0.572 | N/A |  | N/A |  |
| Breastfeeding | 8(50.00) | 24(68.57) | 3(30.00) | 1.83(0.86 to 3.87) | 0.117 | 1.(0.08 to 1.14) | 0.077 | 0.21(0.04 to 1.00) | 0.050 | 0.67(0.12 to 3.81) | 0.649 |
| **Neonatal outcomes** |  |  |  |  |  |  |  |  |  |  |  |
| Congenital malformation | 1(6.25) | 3(8.57) | 0 | N/A | N/A | 1.45(0.14 to 15.16) | 0.755 | N/A |  | N/A |  |
| Preterm birth | 2(12.50) | 5(14.29) | 2(20.00) | 1.25(0.52 to 3.00) | 0.611 | 1.17(0.20 to 6.77) | 0.864 | 1.50(0.24 to 9.22) | 0.662 | 1.32(0.45 to 3.86) | 0.609 |
| Early preterm birth (24-30weeks) | 0 | 2(5.71) | 0 | N/A |  | N/A |  | N/A |  | N/A |  |
| Macrosomia | 0 | 5(14.29) | 1(10.00) | 0.91(0.29 to 2.82) | 0.866 | N/A | N/A | 0.64(0.07 to 6.26) | 0.705 | N/A |  |
| LGA | 1(6.25) | 7(20.00) | 1(10.00) | 0.76(0.25 to 2.29) | 0.631 | 3.89(0.44 to 34.69) | 0.224 | 0.43(0.05 to 3.97) | 0.456 | 1.29(0.31 to 5.48) | 0.729 |
| SGA | 1(6.25) | 4(11.43) | 1(10.00) | 1.20(0.37 to 3.94) | 0.762 | 2.00(0.21 to 19.50) | 0.551 | 0.83(0.08 to 8.43) | 0.877 | 1.29(0.31 to 5.48) | 0.729 |
| Neonatal hypoglycemia | 2(12.50) | 4(11.43) | 0 | N/A | N/A | 0.93(0.15 to 5.72) | 0.941 | N/A |  | N/A |  |
| Neonatal jaundice | 2(12.50) | 8(22.86) | 0 | N/A | N/A | 2.33(0.43 to 12.57) | 0.324 | N/A |  | N/A |  |
| Apgar score < 8 | 2(12.50) | 1(2.86) | 1(10.00) | 1.56(0.44 to 5.53) | 0.493 | 0.13(0.01 to 1.66) | 0.118 | 4.29(0.24 to 77.22) | 0.324 | 0.76(0.21 to 2.78) | 0.674 |
| NICU | 5(31.25) | 5(14.29) | 1(10.00) | 0.67(0.22 to 1.98) | 0.466 | 0.38(0.09 to 1.57) | 0.181 | 0.64(0.07 to 6.26) | 0.705 | 0.49(0.16 to 1.58) | 0.234 |
| RDS | 2(12.50) | 0 | 1(10.00) | N/A |  | N/A |  | N/A |  | 0.88(0.25 to 3.14) | 0.846 |

Data are presented as mean ± standard deviation or N(percentage).

Analysis were adjusted for maternal age, duration of diabetes, BMI, and daily dose of insulin.

NPH, neutral protamine Hagedorn; OR, odds ratio; LGA, large for gestational age; SGA, small for gestational age; NICU, admission to neonatal intensive care unit; RDS, neonatal respiratory distress syndrome.
